# Masquerading Yeast: A Case Report of *Lomentospora prolificans* Fungemia With a Diagnostic Twist

**DOI:** 10.1093/ofid/ofaf598

**Published:** 2025-09-24

**Authors:** Luke A Fenlon, Neeraja Swaminathan

**Affiliations:** Division of Infectious Diseases, Department of Internal Medicine, University of Utah, Salt Lake City, Utah, USA; Division of Clinical Microbiology, Department of Pathology, University of Utah and ARUP Laboratories, Salt Lake City, Utah, USA; Division of Infectious Diseases, Department of Internal Medicine, University of Utah, Salt Lake City, Utah, USA

**Keywords:** conidia, fungemia, *Lomentospora*, neutropenia, *Scedosporium*

## Abstract

*Lomentospora prolificans* is an environmental mold that is increasingly recognized as an opportunistic pathogen, particularly in immunocompromised hosts. Here we present a case of disseminated *Lomentospora* in a patient with refractory acute myeloid leukemia. In general, disseminated mold infections can be challenging to diagnose. The diagnosis in this case was delayed due to a false positive direct from a blood culture multiplex polymerase chain reaction result for *Candida tropicalis* due to amplification of nonviable DNA and misidentification of conidia as yeast forms on gram stain. The *Lomentospora* isolate was resistant to all commercially available antifungal agents, and the patient ultimately succumbed to the infection. This case provides an opportunity to review disseminated *Lomentospora* infections, the associated diagnostic challenges, and limited treatment options.

## CASE PRESENTATION

*Lomentospora prolificans*, formerly classified as *Scedosporium prolificans*, is an environmental fungus that has increasingly been recognized as a highly virulent opportunistic human pathogen [1–3]. Infections occur primarily in immunocompromised hosts but have also been documented in immunocompetent individuals [4]. *L. prolificans* infection is typically acquired via inhalation of conidia or direct inoculation by trauma [5]. Infections range along a spectrum from localized disease to disseminated fungemia. Given the low incidence of disease and overall similarity between organisms, many studies include both *Lomentospora prolificans* and other *Scedosporium* species in a combined analysis. That said, notable differences have been identified. *Lomentospora* is seen more often in patients with underlying hematologic malignancies and has more intrinsic antifungal resistance [1, 6].

In general, diagnosing mold infections such as *Scedosporium* and *Lomentospora* is challenging and requires growth in culture and/or compatible histopathology with associated sequencing data. Certain molds can be isolated from blood cultures, most frequently *Fusarium* followed by *Scedosporium*/*Lomentospora,* and less commonly *Paecilomyces* or *Aspergillus* [7]. Here we present a case of disseminated *Lomentospora prolificans* in a patient with prolonged neutropenia due to refractory acute myeloid leukemia (AML). The case was further complicated by the initial gram stain incorrectly identifying conidia as yeast forms, and direct-from-blood culture multiplex polymerase chain reaction (PCR) yielding a false-positive result of *Candida tropicalis.*

A 56-year-old male with refractory acute myeloid leukemia (AML) was admitted with neutropenic fever to an academic cancer hospital in the mountain west United States of America. His malignancy, initially diagnosed 18 months before his current presentation, had continued to progress despite multiple lines of therapy. After failure of induction therapy (azacitidine with enasidenib), he transitioned to salvage treatment (cladribine, cytarabine, and granulocyte-stimulating factor with mitoxantrone [CLAG-M]) and achieved clinical remission. He declined bone marrow transplant in favor of maintenance oral azacitidine. Four months before his presentation, the patient was found to have disease relapse. At the time of admission, he was on day 23 of treatment with gemtuzumab ozogamicin.

**Figure 1.**
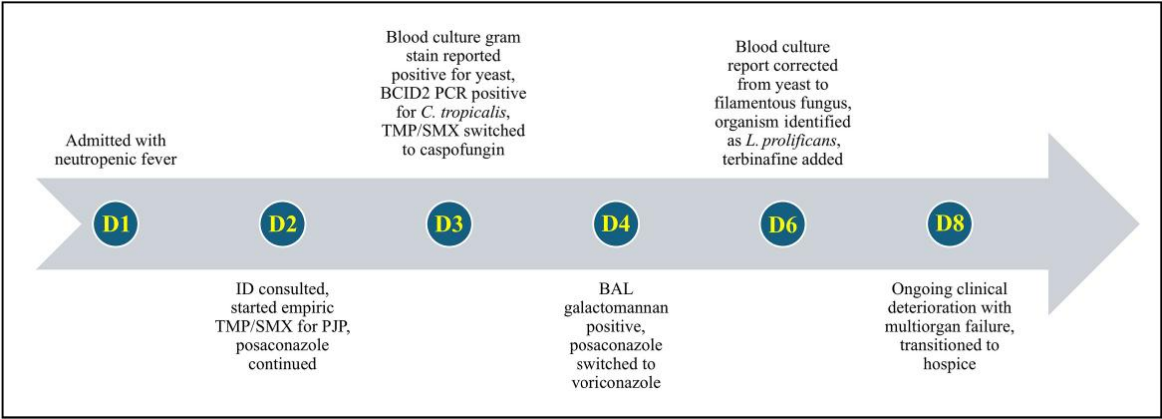
Timeline of key events during hospitalization. Abbreviations: ID, infectious diseases; PCR, polymerase chain reaction; PJP, *Pneumocystis jirovecii*; TMP/SMX, trimethoprim/sulfamethoxazole.

On admission, the patient had been neutropenic for 3 months (Figure 1). He denied any respiratory symptoms including dyspnea, cough, or chest pain but did have a new oxygen requirement of 2-L by nasal cannula. He was febrile to 39.3°C. On pulmonary auscultation, he had intermittent wheezing. The remainder of the exam was unremarkable. Labs were notable for a white blood cell count of 2.87 k/µL with an absolute neutrophil count of 0.3 k/µL, and his serum beta-d-glucan (BDG) was >500 pg/mL. Computed tomography (CT) of the chest demonstrated numerous nodular opacities bilaterally with a cavitary nodule up to 18 mm surrounded with ground glass in the left upper lobe. Before admission, the patient had been taking posaconazole 300 mg daily for antifungal prophylaxis per institutional neutropenia guidelines. The patient did not have recent therapeutic drug monitoring; however, a level checked on admission was within therapeutic range at 2.0. He had also been treated with once-monthly intravenous pentamidine for pneumocystis prophylaxis, with the last dose given 25 days before admission.

At the time of AML diagnosis, incidental lung nodules were discovered on CT chest, prompting bronchoscopy. *Pneumocystis jirovecii* (PJP) PCR was positive, while direct fluorescent antibody, fungal stain, culture, BDG, and histopathology were all negative. Given his lack of respiratory symptoms, PJP colonization was presumed, and no treatment was given.

**Figure 2.**
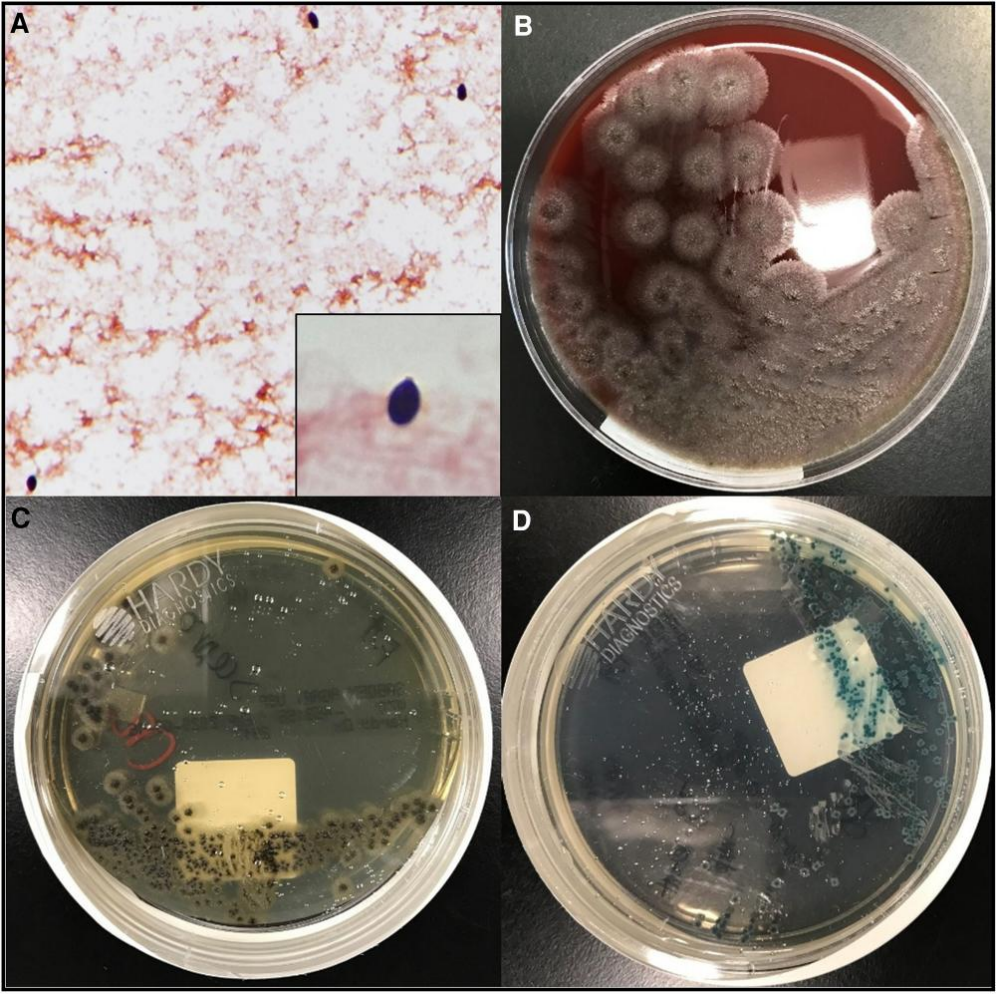
A, Representative field from initial blood culture gram stain demonstrating isolated conidia at 40× magnification. Inset is an enlarged example of a single flask-shaped conidium. Example morphology of a *Lomentospora* isolate growing on (B) sheep blood agar, (C) Sabouraud's dextrose agar, and (D) CHROMagar.

Infectious diseases was consulted on day 2 of admission. Given the elevated serum BDG, history of PJP colonization, and radiological findings, there was concern for PJP pneumonia prompting empiric treatment with trimethoprim-sulfamethoxazole (2 double-strength tablets 3 times daily) in addition to pursuing bronchoscopy and a noninvasive fungal workup. On hospital day 3, the admission blood cultures yielded a preliminary positive result of yeast identified by gram stain (Figure 2). Two hours later, the reflex multiplex PCR was reported positive for *Candida tropicalis*. This test was done using the BioFire BCID2 panel (bioMérieux, Salt Lake City, UT, USA) in a lab using BD BACTEC blood culture bottles. Based on this information, the clinical impression was that the patient had invasive candidiasis, potentially complicated by endocarditis with septic emboli to the lungs. Consequently, caspofungin (150-mg loading dose followed by 100 mg daily) was started, and the patient's chronic indwelling peripherally inserted central venous catheter was removed as this was considered a potential source. Despite this, the patient had ongoing high-grade fevers, increasing oxygen requirements, and progressive renal failure. An elevated BAL *Aspergillus* galactomannan (index 0.96, positive considered >0.5) from bronchoscopy suggested a concomitant pulmonary mold infection. Posaconazole was stopped due to concern for breakthrough and voriconazole started (6 mg/kg every 12 hours for a 2-dose load followed by 4 mg/kg every 12 hours). An *Aspergillus* species PCR on BAL fluid and a PJP PCR were both negative. Three days after the blood culture initially flagged positive for yeast, a correction was issued that the organism was a filamentous fungus and not yeast. On the same day via matrix-assisted laser desorption/ionization–time of flight mass spectrometry, the organism was identified as *Lomentospora prolificans*. As seen in Figure 2B–D, the patient's fungal isolate growth morphology on solid media is consistent with a mold, and no yeast forms are seen. With this new information, terbinafine (500 mg twice daily) was added to voriconazole and caspofungin pending susceptibility data. The BAL fungal culture ultimately grew *Lomentospora* as well. The patient continued to deteriorate and via shared decision-making with the primary team and his family, the patient elected to transition to comfort care. The patient died on hospital day 8. Susceptibility testing of the *Lomentospora* isolate resulted after the patient transitioned to comfort measures. As can be seen in Table 1, this isolate was highly drug resistant, with a minimum inhibitory concentration of amphotericin B ≥8, voriconazole >16, and terbinafine >2.

**Table 1.** Susceptibility Testing of *Lomentospora* Isolate

Antimicrobial Agent	MIC
5-fluorocytosine	≥64
Amphotericin B	≥8
Anidulafungin	4
Caspofungin	≥8
Fluconazole	≥128
Isavuconazole	≥16
Itraconazole	≥16
Ketoconazole	8
Micafungin	≥8
Posaconazole	≥16
Terbinafine	≥2^a^

Abbreviation: MIC, minimum inhibitory concentration.

^a^Performed by nonstandardized methodology.

This case is notable for several reasons. First, disseminated *Lomentospora prolificans* is a rare and highly morbid infection. The patient described in this case had 2 of the most common risk factors for disseminated disease, underlying hematologic malignancy, and prolonged neutropenia [6]. This case also exemplifies a breakthrough invasive fungal infection. Boutin et al. published a systematic review evaluating breakthrough infections in patients with high-risk hematologic disorders on antifungal prophylaxis. In >1000 cases analyzed, *Aspergillus* was the most common breakthrough mold (40% of cases), followed by *Mucor* (20%), *Candida* (18%), and *Fusarium* (9%). *Scedosporium/Lomentospora* was less frequent, accounting for 17 total cases (1.6%) [8].

Research is ongoing to better understand the pathogenicity of *Lomentospora.* One key process driving its virulence is the ability of *L. prolificans* to germinate in vivo, transitioning from conidia to the hyphal form. As hyphae, *Lomentospora* can invade cells and penetrate tissue, including blood vessels. Once established, clearance of infection is inhibited by formation of biofilms. An additional virulence factor is melanin, which protects the fungus from a variety of host insults, including oxidative stress, while also modulating the host immune response by inhibiting phagolysosome formation and host cell apoptosis. Melanin is also responsible for the characteristic pigmentation that can be visualized microscopically [2]. In our patient, we presume the portal of entry was respiratory given compatible CT chest findings and BAL culture growing *L. prolificans*.

Prior analyses looking at outcomes in patients with *Scedosporium* and *Lomentospora* infection have found a survival benefit in patients who have a nidus of infection that can be addressed surgically. To this end, the European Confederation of Medical Mycology and the Australian guidelines for non-*Aspergillus* mold infections both strongly recommend adjunctive surgery when a nidus of infection is amenable to resection [9, 10]. Additional survival benefit has been suggested for combination antifungal therapy with voriconazole and terbinafine. In a retrospective study by Jenks et al., although mortality remained high, voriconazole and terbinafine combination therapy trended toward improved survival [11]. In a study comparing *Scedosporium* with *Lomentospora* infections, neutropenia, disseminated disease, and *L. prolificans* infection were identified as independent predictors of 3-month mortality [6].

This case provides an opportunity to reflect on our diagnostic tools. The utility of serological markers for diagnosis of *Lomentospora* infection is under investigation. In a study by Lamoth et al. analyzing 27 immunosuppressed patients with invasive scedosporiosis or lomentosporiosis, serum BDG had an 81.5% sensitivity for invasive infection [12]. Serum BDG in this patient was >500 pg/mL. Ongoing research is evaluating alternative diagnostics more specific to individual fungal species. Thorton et al. have isolated antibodies specific to *L. prolificans* that target tetrahydroxy naphthalene reductase, an enzyme in the melanin biosynthetic pathway [13].

In our patient, the initial blood culture microscopy was reported as yeast, whereas in hindsight it was *Lomentospora* conidia. During infection, *L. prolificans* can exist in both branching hyphae and conidia forms. The hyphal form is typically found in tissue and microscopically noted to be septate with irregular branching. Conidia are flask shaped and measure 2–5 × 3–12 µm [14]. Conidiation can occur within the host and is thought to be a driving factor for intravascular dissemination. Both yeast and hyphal structures can be visualized on gram stain. Distinct from *Scedosporium*, *L. prolificans* can produce melanin and appear highly pigmented. Of note, *Candida* species are spherical yeast forms that can be similar in size. Given similarities of size and shape between conidia and yeast, one can see how these structures may appear similar in isolation under microscopy. Figure 2A is a representative field of a gram stain from the patient's blood culture demonstrating isolated conidia. Growth of the organism is also demonstrated on plates; notably the colony morphology is consistent with mold. Also, the dark pigmentation in Figure 2C is consistent with *Lomentospora*. This case demonstrates the importance of understanding the life cycle of fungal pathogens and what structures are expected to be visualized from patient blood or tissue samples.

Our case was further confounded by a false-positive result on the BCID2 PCR panel. Before this event, there had been a Food and Drug Administration (FDA) Class 2 recall for the BCID2 PCR panel when used with BD BACTEC blood culture vials due to amplification of nonviable *C. tropicalis* DNA resulting in potential false-positive results [15]. When the result for our patient was reported by the performing laboratory, a disclaimer was included: “*Candida tropicalis* could be a potential contaminant. Interpret results in conjunction with other clinical symptoms.” In this case with a serum BDG above the limit of detection and yeast reported on gram stain, the clinical context was plausibly disseminated candidemia. This is not the first example of this phenomenon; there have been similar reports in the past with *Pseudomonas aeruginosa*, *Enterococcus*, and *Proteus* DNA being detected in the absence of viable organisms [16, 17]. After the recall, the manufacturing company filed a corrective action software update targeted at mitigating the false-positive result. As nucleic acid amplification is increasingly being used as a diagnostic technique, the limitations need to be acknowledged and clinical relevance taken into consideration when making a diagnosis.

Ultimately, it is unlikely that expedited identification (eg, mold called on initial gram stain) would have changed the ultimate outcome, as the patient's condition deteriorated rapidly with multiorgan failure and the *Lomentospora* isolate was resistant to guideline-directed empiric treatment of voriconazole and terbinafine (Table 1). As mentioned previously, *L. prolificans* is known to be intrinsically multidrug resistant. Research is ongoing into the efficacy of new antifungal agents such as olorofim, fosmanogepix, and ibrexafungerp. Preliminary studies are promising for olorofim and suggest up to 80 times more activity than current commercially available agents. Phase III studies are ongoing [2]. Duration of therapy has been suggested at 4–6 months depending on overall response to treatment. As an augmentation to antifungal therapy, studies have looked at other immunomodulatory agents such as granulocyte colony-stimulating factor (G-CSF). Work in animal models suggests that G-CSF may improve survival outcomes. Use has been documented in other case reports of successful treatment [9].

We have presented a case of disseminated *L. prolificans* in an immunocompromised host with refractory AML. This case adds to the growing body of literature regarding *Lomentospora* infections and serves as a reminder of the need for both improved diagnostics and novel antifungal treatment for this emerging pathogen.

## References

[1] Konsoula A, Tsioutis C, Markaki I, Papadakis M, Agouridis AP, Spernovasilis N. *Lomentospora prolificans*: an emerging opportunistic fungal pathogen. Microorganisms 2022; 10:1317. 35889036 10.3390/microorganisms10071317 PMC9316904

[2] Neoh CF, Chen SC, Lanternier F, et al Scedosporiosis and lomentosporiosis: modern perspectives on these difficult-to-treat rare mold infections. Clin Microbiol Rev 2024; 37:e0000423. 38551323 10.1128/cmr.00004-23 PMC11237582

[3] Konsoula A, Agouridis AP, Markaki L, Tsioutis C, Spernovasilis N. *Lomentospora prolificans* disseminated infections: a systematic review of reported cases. Pathogens 2023; 12:67. 10.3390/pathogens12010067 PMC9861501 36678415

[4] Cortez KJ, Roilides E, Quiroz-Telles F, et al Infections caused by *Scedosporium* spp. Clin Microbiol Rev 2008; 21:157–97. 18202441 10.1128/CMR.00039-07 PMC2223844

[5] Kermani F, Yazdani Charati J, Roohi B, et al A systematic review and disability-adjusted life years of *Scedosporium/Lomentospora* infection in patients after near-drowning. Mycoses 2024; 67:e13703. 38345265 10.1111/myc.13703

[6] Bronnimann D, Garcia-Hermoso D, Dromer F, et al Scedosporiosis/lomentosporiosis Observational Study (SOS): clinical significance of *Scedosporium* species identification. Med Mycol 2021; 59:486–97. 33037432 10.1093/mmy/myaa086

[7] Zhang SX, Babady NE, Hanson KE, et al Recognition of diagnostic gaps for laboratory diagnosis of fungal diseases: expert opinion from the Fungal Diagnostics Laboratories Consortium (FDLC). J Clin Microbiol 2021; 59:e0178420. 33504591 10.1128/JCM.01784-20 PMC8218742

[8] Boutin CA, Durocher F, Beauchemin S, Ziegler D, Abou Chakra CN, Dufresne SF. Breakthrough invasive fungal infections in patients with high-risk hematological disorders receiving voriconazole and posaconazole prophylaxis: a systematic review. Clin Infect Dis 2024; 79:151–60. 38752732 10.1093/cid/ciae203 PMC11259221

[9] Bupha-Intr O, Butters C, Reynolds G, et al Consensus guidelines for the diagnosis and management of invasive fungal disease due to moulds other than *Aspergillus* in the haematology/oncology setting, 2021. Intern Med J 2021; 51:177–219. 34937139 10.1111/imj.15592

[10] Hoenigl M, Salmanton-García J, Walsh TJ, et al Global guideline for the diagnosis and management of rare mould infections: an initiative of the European Confederation of Medical Mycology in Cooperation With the International Society for Human and Animal Mycology and the American Society for Microbiology. Lancet Infect Dis 2021; 21:e246–57. 33606997 10.1016/S1473-3099(20)30784-2

[11] Jenks JD, Seidel D, Cornely OA, et al Voriconazole plus terbinafine combination antifungal therapy for invasive *Lomentospora prolificans* infections: analysis of 41 patients from the FungiScope® registry 2008–2019. Clin Microbiol Infect 2020; 26:784.e1–e5. 10.1016/j.cmi.2020.01.012 31972317

[12] Lamoth F, Nucci M, Fernandez-Cruz A, et al Performance of the beta-glucan test for the diagnosis of invasive fusariosis and scedosporiosis: a meta-analysis. Med Mycol 2023; 61:myad061. 37381179 10.1093/mmy/myad061 PMC10405209

[13] Thornton CR, Ryder LS, Le Cocq K, Soanes DM. Identifying the emerging human pathogen *Scedosporium prolificans* by using a species-specific monoclonal antibody that binds to the melanin biosynthetic enzyme tetrahydroxynaphthalene reductase. Environ Microbiol 2015; 17:1023–38. 24684242 10.1111/1462-2920.12470

[14] Chen SCA, Halliday CL, Hoenigl M, Cornely OA, Meyer W. *Scedosporium* and *Lomentospora* infections: contemporary microbiological tools for the diagnosis of invasive disease. J Fungi 2021; 7:23. 10.3390/jof7010023 PMC7823285 33406673

[15] US Food & Drug Administration. Class 2 device recall BIOFIRE Blood Culture Identification 2 BCID2 panel. **2024**. Available at: https://www.accessdata.fda.gov/scripts/cdrh/cfdocs/cfres/res.cfm?id=204997#:~:text=On%2001%2F26%2F2024%2C,the%20bottle%20types%3A%20BD%20BACTEC%22. Accessed 20 May 2025.

[16] US Food & Drug Administration. Class 2 device recall FilmArray BCID panel. **2019**. *A*vailable at: https://www.accessdata.fda.gov/scripts/cdrh/cfdocs/cfRes/res.cfm?ID=171432. Accessed 20 May 2025.

[17] US Food & Drug Administration. Class 2 device recall FilmArray Blood Culture Identification (BCID) panel. **2014**. Available at: https://www.accessdata.fda.gov/scripts/cdrh/cfdocs/cfRES/res.cfm?id = 127466. Accessed 20 May 2025.

